# Chromosome-scale assembly of the yellow mealworm genome

**DOI:** 10.12688/openreseurope.13987.2

**Published:** 2022-02-25

**Authors:** Evangelia Eleftheriou, Jean-Marc Aury, Benoît Vacherie, Benjamin Istace, Caroline Belser, Benjamin Noel, Yannick Moret, Thierry Rigaud, Fabrice Berro, Sona Gasparian, Karine Labadie-Bretheau, Thomas Lefebvre, Mohammed-Amin Madoui

**Affiliations:** 1 Génomique Métabolique, Genoscope, Institut François Jacob, Commissariat à l'Energie Atomique (CEA), CNRS, Univ Evry, Université Paris-Saclay, Université Paris-Saclay, Evry, 91057, France; 2 Genoscope, Institut de biologie François Jacob, CEA, Université Paris‐Saclay, Evry, 91057, France; 3 Équipe Écologie Évolutive, UMR CNRS 6282 BioGéoSciences, Université de Bourgogne Franche-Comté, Dijon, 21000, France; 4 Ynsect, Evry, 91000, France; 5 Service d’Etude des Prions et des Infections Atypiques (SEPIA), Institut François Jacob, Commissariat à l’Energie Atomique et aux Energies Alternatives (CEA), Université Paris Saclay, Fontenay-aux-Roses, France

**Keywords:** Yellow Mealworm, Tenebrio molitor, genomics, chromosome-scale assembly

## Abstract

**Background:** The yellow mealworm beetle, *Tenebrio molitor*, is a promising alternative protein source for animal and human nutrition and its farming involves relatively low environmental costs. For these reasons, its industrial scale production started this century. However, to optimize and breed sustainable new *T. molitor* lines, the access to its genome remains essential.

**Methods: **By combining Oxford Nanopore and Illumina Hi-C data, we constructed a high-quality chromosome-scale assembly of *T. molitor*. Then, we combined RNA-seq data and available coleoptera proteomes for gene prediction with GMOVE.

**Results:** We produced a high-quality genome with a N50 = 21.9Mb with a completeness of 99.5% and predicted 21,435 genes with a median size of 1,780 bp. Gene orthology between *T. molitor* and *Tribolium castaneaum* showed a highly conserved synteny between the two coleoptera and paralogs search revealed an expansion of histones in the *T. molitor* genome.

**Conclusions:** The present genome will greatly help fundamental and applied research such as genetic breeding and will contribute to the sustainable production of the yellow mealworm.

## Plain language summary

We provide the genome sequence of the yellow mealworm, *Tenebrio molitor*, by combining high-throughput sequencing technologies to obtain a genome assembly that well represents the 10 mealworm chromosomes. We also identified the *Tenebrio molitor* gene set and compared its organisation to that of the red beetle. This new genomic resource will help breeders to develop new mealworm lines to face future global human nutrition problems by providing protein-rich and ecologically friendly mealworm production systems.

## Introduction

The global human population is estimated to reach approximately nine billion people by 2050, thus the demand for animal protein is expected to increase by 76% ^1^. Such an increase questions the sustainability of our conventional food and feed production systems. At the same time, we also need to reduce the impact of agriculture on our environment ^2^. Today, insect production is considered a sustainable alternative for food and feed production for several reasons. First, the suitable nutritional composition of edible insects ^3^ and second, the relatively low environmental impact its production involves compared to other conventional livestock production systems ^4, 5^.

In this context, the yellow mealworm beetle *Tenebrio molitor* has been described as a promising alternative protein source for animal and even human nutrition ^6^. For these reasons several companies have pioneered the production of *T. molitor* at industrial scale. However, despite being promising for sustainable food security, mass production of *T. molitor* remains relatively primitive and challenging ^7^.

The genetic improvement of *T. molitor* is one of these challenges. Indeed, several quantitative traits of industrial importance such as growth rate, fertility, protein rate or susceptibility to pathogens need to be mapped to allow the development of molecular-based breeding programs to speed up the development of new lines with improved agronomic traits. However, suitable genomic resources on *T. molitor* are needed to accelerate such genetic programs.

Previous efforts to produce *T. molitor* transcriptomes and more recently the draft genome using 10X genomics technology have been published ^8^. While this latter technology was promising on diploid and heterozygous insects ^9– 12^, its application to *T. molitor* produced a fragmented assembly with a 90% of BUSCO completeness and no genome annotation. This particular effort motivated the development of a new genome assembly that would allow deeper genomic analyses such as quantitative trait locus mapping or genomic estimated breeding values analysis.

Here, we present a *T. molitor* genome assembly based on the combination of long, short reads and Hi-C data. The genome assembly and annotation quality are analysed and a comparison to the red flour beetle ( *Tribolium castaneum*) genome is described to show how the current genome can be an asset for academic research and breeding.

## Methods

### Biological material and insect rearing

*Tenebrio molitor* samples were provided by Ynsect and bred at CEA-Genoscope (Evry, France). The individuals were fed with bran and apple and kept at room temperature and humidity. For the genome sequencing, male nymphs which possess XY chromosomes were selected, starved for three days and used for DNA extraction. For mRNA extraction, eggs, larva, nymphs, adult males and females were isolated without specific diet.

### DNA extraction

Genomic DNA (gDNA) was extracted from a single nymph male to generate both Illumina PCR-free, PromethION and Dovetail Hi-C libraries. In order to generate long reads on the Oxford Nanopore Technologies devices, high-quality and high-molecular-weight (HMW) DNA was needed. For this purpose, DNA was isolated following the protocol provided by Oxford Nanopore Technologies, Oxford, UK (ONT), “High molecular weight gDNA extraction from plant leaves” provided by the ONT Community in March, 2019 (CTAB-Genomic-tip). This protocol involves a conventional CTAB extraction followed by purification using commercial Qiagen Genomic tips (QIAGEN, MD, USA). DNA fragment size selection was performed using the Short Read Eliminator (Circulomics, MD, USA) instead of AMPpure XP beads. A single nymph male weighing 170mg was cryoground in liquid nitrogen. The fine powder was divided in one-third for the Hi-C library and two-thirds for both Illumina PCR-free and PromethION libraries. The two-thirds of the powder was transferred to a lysis Carlson buffer supplemented with RNase A. After 1h-incubation, proteins were removed with chloroform centrifugation and DNA was precipitated with isopropanol and centrifugation. The pellet was then purified using the Qiagen Genomic tip 100/G, following the manufacturer’s instructions. DNA was quantified by a dsDNA-specific fluorimetric quantitation method using Qubit dsDNA HS Assays (Catalog #Q32851, ThermoFisher Scientific, Waltham, MA). HMW gDNA quality was checked on a 2200 TapeStation automated electrophoresis system (Agilent, CA, USA) and the length of the DNA molecules was estimated to be over 60Kb.

### PromethION library preparation and sequencing

HMW gDNA was size-selected using the Short Read Eliminator kit (SKU SS-100-101-01, Circulomics, MD, USA). The ONT library was prepared with the Oxford Nanopore SQK-LSK109 kit, according to the following protocol. Genomic DNA fragments (3 *µ*g) were repaired and 3'-adenylated with the NEBNext FFPE DNA Repair Mix (Catalog#M6630, New England Biolabs, Ipswich, MA, USA) and the NEBNext® Ultra™ II End Repair/dA-Tailing Module (Catalog#E7546, NEB). Sequencing adapters provided by ONT were ligated using the NEBNext Quick Ligation Module (Catalog#E6056, NEB). After purification with AMPure XP beads (Beckmann Coulter, Brea, CA, USA), half of the library was mixed with the Sequencing Buffer (ONT) and the Loading Bead (ONT) and loaded on a PromethION R9.4.1 flow cell. The second half of the library was loaded on the flow cell after a Nuclease Flush using the Flow Cell Wash Kit (Catalog#EXPWSH003, ONT) according to the ONT protocol. After 48h of the sequencing run, a second Nuclease Flush was performed and a third library was loaded on the flow cell. Nucleotide bases were called using Guppy version 4.0.1 ^13^ and the raw reads were used for genome assembly.

### Illumina PCR-free library preparation and sequencing

The PCR-free library was prepared using the Kapa Hyper Prep Kit (Catalog#KK8505, KapaBiosystems, Wilmington, MA, USA), following the manufacturer’s recommendations. Briefly, qDNA (1.5 *µ*g) was sonicated to a 100–1,500-bp size range using a Covaris E220 sonicator (Covaris, Woburn, MA, USA). The fragments were end-repaired, then 3'-adenylated and Illumina adapters were added. The ligation products were purified with AMPure XP beads (Beckmann Coulter Genomics, Danvers, MA, USA). The library was quantified by qPCR using the KAPA Library Quantification Kit for Illumina Libraries (Catalog#07960140001, KapaBiosystems), and the library profiles were assessed on an Agilent 2100 Bioanalyzer (Agilent Technologies, Santa Clara, CA, USA). The libraries were sequenced on an Illumina HiSeq4000 instrument (Illumina, San Diego, CA, USA) using 150 bp read chemistry in paired-end mode. After the Illumina sequencing, an in-house quality control process was applied to the reads that passed the Illumina quality filters, as described by Alberti and colleagues ^14^.

### Dovetail Hi-C library preparation and sequencing

Another third of the cryoground powder (from the DNA extraction section) was used to generate a Hi-C library using the Dovetail Hi-C preparation kit (Dovetail Genomics, Scotts Valley, CA, USA), according to the manufacturer’s protocol (manual version 1.03). After the cross-linking of animal tissues, the chromatin was normalized and then immobilized on capture beads before enzyme restriction digestion. The digested DNA ends were marked with biotin and ligated to create chimeric molecules. After reversal cross-linking, DNA was purified and then followed by library generation. The Dovetail Hi-C library quality was checked as described above and sequenced on an Illumina HiSeq4000 instrument (Illumina, San Diego, CA, USA) using 150 base-length read chemistry in paired-end mode.

### RNA extraction

Eggs, larva, nymphs, adult males and females were collected for later mRNA extraction. Tissue samples were mechanically homogenized using ZR Bashing Bead Lysis tube (ZymoResearch, CA, USA) with the FastPrep-24™ 5G Instrument (MP Biomedicals, Santa Ana, CA, USA). Nucleic acids were then extracted from homogenized suspension using the ZR-Duet DNA/RNA MiniPrep Plus kit (Catalog # D7003, ZymoResearch, CA, USA). Extracted RNA was quantified with RNA-specific fluorometric quantitation on a Qubit 2.0 Fluorometer using Qubit RNA HS Assay (Thermo Fisher Scientific, Waltham, MA, USA). Integrity of total RNA was assessed on an Agilent Bioanalyzer, using the RNA 6,000 Pico LabChip kit (Catalog # 5067-1513, Agilent Technologies, Santa Clara, CA).

### RNA library preparation and sequencing

RNA-seq library preparations were carried out from 500ng total RNA using the TruSeq Stranded mRNA kit (Catalog #20020595, Illumina, San Diego, CA, USA), which allows mRNA strand orientation, i.e sequence reads occur in the same orientation as antisense RNA. Poly(A)+ RNA was selected with oligo(dT) beads, chemically fragmented and converted into single-stranded cDNA using random hexamer priming. Then, the second strand was generated to create double-stranded cDNA. cDNA were then 3'-adenylated, and Illumina adapters were added. Ligation products were PCR-amplified. Ready-to-sequence Illumina libraries were then quantified by qPCR using the KAPA Library Quantification Kit for Illumina Libraries (Catalog #KK4824, KapaBiosystems, Wilmington, MA, USA), and library profiles evaluated with an Agilent 2100 Bioanalyzer (Agilent Technologies, Santa Clara, CA, USA). Each library was sequenced using 151bp paired end reads chemistry on a NovaSeq 6000 Illumina sequencer.

### Genome assembly

The *T. molitor* genome size was estimated using GenomeScope ^15^ (GenomeScope, RRID:SCR_017014) v1 with Illumina reads (Table S1, *Extended data*) and a *k*-mer value of 31. We applied YACRD ^16^ (version 0.6.0) to the raw nanopore reads (Table S1, *Extended data*) to detect potential chimeras. Both "all-vs-all alignment" and "yacrd scrubbing" steps were performed with the recommended parameters and removed 109,066 chimeric reads. The 2,372,861 non-chimeric reads were corrected using NECAT ^17^ with parameters GENOME_SIZE, PREP_OUTPUT_COVERAGE and CNS_OUTPUT_COVERAGE set to 310,000,000, 60 and 40, respectively, to first correct the longest 60x reads and afterwards, the longest 40x corrected reads were extracted to assemble 250,277 reads.

Because nanopore reads contain systematic errors in homopolymeric regions, the output assembly was polished three times using Racon ^18^ (Racon, RRID:SCR_017642) with default parameters with the nanopore reads and two times Hapo-G ^19^ with the Illumina reads. The assembly was merged into a single haplotype genome assembly using HaploMerger2 ^20^ (Figure S2, *Extended data*) and polished using two rounds of Hapo-G with the Illumina reads.

To increase the contiguity of the assembly to a chromosome-scale level (Table S1, *Extended data*), we aligned Hi-C paired-end reads to the polished haploid assembly with *bwa − mem* ^21^ (BWA, RRID:SCR_010910). Because Hi-C captures conformation via proximity-ligated fragments, paired-end reads are first mapped independently (as single-end reads) and subsequently paired in a later step. Hi-C reads and alignments contain experimental artifacts so the alignments need some additional processing. We use alignment filtering method using Arima Genomics pipeline ( https://github.com/ArimaGenomics/mapping_pipeline) and applied the script "filter l" to each bam file (Read1 and Read2) and afterwards paired the filtered single-end Hi-C reads using "two_read_bam_combiner.pl". Then, with Picard tools ( https://broadinstitute.github.io/picard/), we added read groups to the combined BAM file (with the command AddOrReplaceReadGroups) and discarded any PCR duplicates present in the paired-end BAM file (with the command MarkDuplicates). We scaffolded the assembly with the Hi-C data using SALSA2 ^22^ and obtained 138 scaffolds. Only scaffolds larger than 35kb were kept resulting in a final assembly of 112 scaffolds (Table S3 and Figure S10, *Extended data*). The largest scaffolds were manually checked for missassembly using the sequencing information and the synteny with the ten *Tribolium castaneum* chromosomes (cf. comparative genomics section).

### Transcriptome assembly

RNA-seq reads from six transcriptomes derived from different developmental stages (larvae, pupae, juveniles, adults), sexes (females and males) and public data of two RNA-seq samples generated from pooled bacterial infected *T. molitor* ( PRJNA646689, *Underlying data*) were assembled using Velvet ^23^ (Velvet, RRID:SCR_010755) version 1.2.07 and Oases ^24^ (Oases, RRID:SCR_011896) version 0.2.08 with *k*-mer size set to 81 and 63 for the in-house and public RNA-seq reads, respectively (Table S2, *Extended data*). The first five bases of contigs 5' and 3' ends were removed. The sequences were masked for low-complexity using DustMasker (version 1.0.0 from the BLAST 2.10.0 package) and only contigs larger than 150bp with more than 75% of unmasked bases were kept. To address the problem of merged chimeric contigs, a post-processing of Oases contigs has been done. Assembly tools often erroneously merge sequences into one single contig and Oases is prone to this behaviour. To address this problem, we used an in-house script that splits chimeric contigs. Splitting a contig into regions where different ORFs appear, or regions where abrupt shifts in read coverage occur, could streamline the gene-prediction process. Based on combined resources such as the pileup-coverage, the research of ORFs (TransDecoder https://github.com/TransDecoder/TransDecoder/releases) and domains, this tool aims to split contigs sequences with different functional sites form different contigs. Reads were mapped to the contigs with BWA-mem and the consistent paired-end reads were selected. Chimeric contigs were identified and split (uncovered regions) based on coverage information from consistent paired-end reads. Moreover, open reading frames (ORF) and domains were searched using respectively TransDecoder and CDDsearch (Conserved Domain Database, RRID:SCR_002077). We only allowed breaks outside ORF and domains. Finally, the read strand information was used to correctly orient the RNA-seq contigs.

### Genome annotation

***Repeated sequence masking.*** Low complexity regions of the assembly were masked with the DustMasker ^25^ algorithms (version 1.0.0 from the BLAST 2.10.0 package). Transposable elements (TEs) and other repeats were annotated and masked using RepeatMasker ^26^ (RepeatMasker, RRID:SCR_012954) version open-4.0.5 with rmblastn ^26^ version 2.10.0+. The assembly was compared to classified sequences of the RepeatMasker complete database 20150807. We set the custom library RepeatMasker.lib of version 4.0.5 to the -lib parameter ^27^.

***Transcriptome and proteome alignments.*** mRNA contigs from the eight samples were aligned to the assembly in a two-step strategy. First, BLAT ^28^ (BLAT, RRID:SCR_011919) (version 36 with default parameters) was used for fast localizing genomic regions and the best match of each contig was kept. A second local alignment was performed with Est2Genome ^29^ (version 5.2 with default parameters). Aligned contigs with overlap higher than 80% and more than 95% identity were retained. Additionally, proteomes of four other Coleoptera ( *T. castaneum*, *Ontophagus taurus*, *Asbolus verrucosus*, *Dendroctonus ponderosae*) and *T. molitor* proteins from UniProt ^30^ database were aligned to the genome in a two-step strategy. First, using BLAT (version 36 with default parameter) matches with score higher than 90% of the best match score were retained. Second, alignments were refined using Genewise ^31^ (version 2.2.0 default parameters) and proteins with more than 50% of their length aligned onto the assembly were kept.

***Gene predictions.*** To identify the gene structure, the transcriptomic and protein alignments were combined using Gmove ^32^ (Gmove, RRID:SCR_019132) (Note S2, *Extended data*). Protein alignments from the five coleoptera were merged into a single file and provided to Gmove (–prot parameter). We also set transcriptomic alignments from eight different samples (Table S2, *Extended data*) to the –rna parameter and activated the –score option to keep the gene model with the highest score. Based on *T. castaneum* gene features, we set the maximal size of intron and minimal size of exons to 150,000bp and 3bp using the -m and -e parameters, respectively. To prevent false positive gene predictions due to a large number of single-exon transcripts, sample-specific single-exon transcripts were removed before running Gmove.

Several criteria were applied sequentially to filter the gene predictions. We used HMMER ^33^ (Hmmer, RRID:SCR_005305) (version 3.2.1, June 2018) to find pfam domains, DIAMOND ^34^ (DIAMOND, RRID:SCR_016071) version 0.9.24 for protein searches against ncbi-nr database, RepeatModeler ^35^ (RepeatModeler, RRID:SCR_015027) version 2.0.1 for *ab initio* repeats screening and TransposonPSI ^36^ to compare predicted models with transposable elements. Gmove initially predicted 24,870 genes, 27% of which were intronless. While we are more confident in multi-exon gene predictions, we were cautious with intronless genes corresponding potentially to transposable elements or false positive predictions caused by the fragmented alignments of transcripts. To solve this problem, we launched HMMER (version 3.2.1 with e-value set to 10e-5) for detecting known pfam domains and a DIAMOND analysis against ncbi-nr database for protein hits (version 0.9.24 with – evalue 10e-5, –unal 0). Furthermore, Repeat-Modeler version 2.0.1 was used for screening *ab initio* repeats in the *T. molitor* assembly and 46.77% of the genome was masked. Then, we focused on overlaps between the predicted genes and repeats, using commands from BEDtools. Genes with exons highly covered by repeats (>90%) were automatically classified as repeats. In parallel, we used transposonPSI.pl to align the virtual cDNA proteins of the 24,870 predictions against the TransposonPSI_08222010 library. We selected the single best transposonPSI match for each protein (from file proteins.fasta.TPSI.topHits) and tagged the corresponding genes as transposable elements. At this point, we excluded genes (single and multi exon) that were either highly covered by repeats (RepeatModeler) or TE tagged (TransposonPSI) without any blastp/pfam hit. We also excluded intronless genes that were predicted only by RNA-seq evidence (not any Coleoptera protein overlap) and at the same time composed of >80% untranslated regions (ratio UTR/(UTR+CDS)) without any pfam/blastp hit.

Additionally, we searched for overlaps between predicted intronless genes and CDS of protein or mRNA evidence. Then, we discarded any intronless gene accomplishing none of the following conditions: (i) A gene that is predicted from at least one mRNA and one protein evidence. (ii) A gene that is predicted from mRNA transcripts of at least two different samples and (iii) A gene that is predicted from at least a *T. molitor* protein (from Uniprot). If none of the above criteria was met and a gene did not have any pfam/blastp hit either, then it was removed. After this filtering process the different annotation supports were combined to obtain a final set of 21,435 gene predictions (see Figure S11, *Extended data* for the genome annotation workflow).

### Comparative genomics

Homology search between the 21,435 *T. molitor* predicted genes and the 22,610 *T. castaneum* protein isoforms was performed. We used blastp ^37^ (NCBI BLAST, RRID:SCR_004870) v.2.10.0+ with a maximum e-value set to 1e-10 and found 10,495 reciprocal best hits between the two species. Using NUCmer from the MUMmer4.0beta ^38^ (MUMmer, RRID:SCR_018171) package, we plotted the alignments between the 16 longest *T. molitor* scaffolds and the 10 *T. castaneum* chromosomes. To observe the synteny between the two beetle genomes, we combined the associations inferred from the MUMmer plot with the localization of the orthologous genes and constructed a Circos plot ^39^ (Circos, RRID:SCR_011798). Finally, 9,760 reciprocal best matches out of the total best hits (10,495) corresponded to orthologous genes between the 16 *T. molitor* scaffolds and the 10 *T. castaneum* chromosomes.

## Results and discussion

### Tenebrio molitor chromosome-scale genome assembly

The *T. molitor* genome size was estimated around 310 Mb with a heterozygosity rate of 1.43% (Figure S1, *Extended data*). By combining long, short reads and Hi-C data, we obtained a final genome assembly of 287.9 Mb ( Table 1) representing a single haplotype of the *T. molitor* diploid genome (2n=20) ^40^ with a BUSCO ^41^ (BUSCO, RRID:SCR_015008) completeness of 99.5% (using version 5.0.0 with Insecta database odb10) (Figure S1, *Extended data*). The assembly presents a N50 of 21.9Mb, which is higher than *T. castaneum*’s one ^42^ and much higher than the previously published *T. molitor* genome N50 (24.1kb). In our assembly, the largest 16 scaffolds represent 90% of the total assembly, leading to a chromosome-scale assembly which provides high-quality support for gene annotation.

**Table 1.** Assembly and BUSCO Metrics for *T. molitor* (versions 2020, 2021) and *T. castaneum*.

Assembly statistics	Tenebrio 2021	Tenebrio 2020	Tribolium
# Contigs	112 (110 nuclear + 2 mitochondrial)	31,390	2,082
Cumulative size	287,931,689	280,780,514	165,944,485
Max contig length	33,042,542	271,822	31,381,287
Mean contig length	2,570,819	8,945	79,704
N50 (L50)	21,885,684 (6)	24,131 (3,180)	15,265,516 (5)
N90 (L90)	5,674,206 (16)	3,289 (16,525)	885,624 (12)
auN	18,643,178	30,387	15,592,941
GC%	36.72%	36.03%	33.86%
Number of N	28,500 (0.01%)	0 (0.00%)	13,515,130 (8.14%)
**BUSCO on genome (N = 1,367)**			
Complete	1,360 (99.5%)	1,213 (88.7%)	1,357 (99.2%)
Duplicated	7 (0.5%)	52 (3.8%)	6 (0.4%)
Fragmented	3 (0.2%)	67 (4.9%)	5 (0.4%)
Missing	4 (0.3%)	87 (6.4%)	5 (0.4%)

Nearly 6% of the assembly was masked for repeated elements with a majority of simple DNA repeats (49,992) and transposons (29,182). The next most abundant repeats were long interspersed nuclear elements (11,417) followed by long terminal repeats (7,950). Overall, these four types of repeats account for 5.31% of the masked genome assembly (Table S4, *Extended data*).

Several studies pointed out the presence of a 142bp satellite highly present in the *T. molitor* genome ^8, 43, 44^. RepeatMasker detected 406 instances of the satellite repeats across 26 scaffolds covering up to 248,412 bp (or 0.08% of the assembly). Additionally, we performed a BLAST analysis with more stringent alignment parameters (BLASTn overlap >80%, identity ≥90%) and the satellite was newly detected in 17 scaffolds. We also found two variant sequences of this satellite (blastn evalue *≤*10e-5, word_size=10) highly represented in scaffold 23. The longest form covers approximately 89% of the satellite (126-129bp), with average identity score 77%, while the shorter one, which is more abundant, covers about 44% of satellite (62-66bp) with mean sequence similarity of 85% (Figure S5, *Extended data*).

The mitochondrial genome was detected in two scaffolds. More precisely, the genbank mitochondrial genome of *T. molitor* (15,785 bp) was aligned to our assembly using Minimap2 ^45^ and detected three times in scaffold 94 with a nucleotide identity of 85–89% (Figure S6, *Extended data*) but also in several other regions of the same scaffold with a lower nucleotide identity (53–73%) (Table S5, *Extended data*). The high copy number of mitochondrial DNA (mtDNA) per cell leads to relatively high depth of coverage, which causes misassemblies. NECAT constructed initially one single contig presenting three supplicated mitochondrial genomes. To resolve this misassembly, we re-assembled long reads that aligned to scaffold 94, using Flye version 2.9 (Flye, RRID:SCR_017016) with genome size parameter set to 15k. Subsequently, the mitogenome was polished using Racon and Hapo-G with short reads (with the same methods used for the whole genome assembly) and obtained one single contig of 15,724 bp. The latter aligns with 98.39% identity to the *T. molitor* genbank mitogenome (15,785 bp) (Figure S8). Mitochondrial DNA was also detected in scaffold 65 (Figure S7, Extended data). However, due to its low ANI (50–75%) and the small fraction it occupies in the scaffold, we considered this alignment as a probable insertion of mtDNA in the nuclear genome. In view of the above considerations, we kept scaffold 65 in the current nuclear genome assembly and removed scaffold 94 as the mitochondrial genome.

### Tenebrio molitor genome annotation

By combining RNA-seq and Coleoptera proteomes, we predicted a total of 21,435 genes which is higher than the number observed in *T. castaneum*. Beside this difference, other metrics are very comparable ( Table 2). Quality of the gene prediction was assessed using BUSCO version 5.0.0 with Insecta database odb10 which contains 1,367 genes and showed a gene completeness of 96.5%. The published gene prediction based on the *T. castaneum* genome has fewer genes but a higher BUSCO score, which reflects the completeness of the gene prediction. The tools and resources (transcriptomes, proteomes) used for gene prediction are different for the two beetles, so we can expect different gene completion between the two predictions. However, the observed difference in the number of predicted genes may rather refer to gene evolution, for example through gene duplication as further explained. 

**Table 2.** Annotation and BUSCO metrics for *T. molitor* 2021 and *T. castaneum*.

Annotation Statistics	Tenebrio 2021	Tribolium
Number of genes (without isoforms)	21,435	14,503
Number of intronless genes	4,898	1,109
Gene length (mean : median)	7,590 : 1,779	8,032 : 2,364
Gene length without UTR (mean : median)	5,785 : 1,147	7,900 : 2,341
Number of exons per gene (mean : median)	4.15 : 3	5.19 : 4
Number of exons per gene (mean : median) Restricted to multi-exon genes	5.08 : 4	5.54 : 4
CDSs length (mean : median)	1,177 : 783	1,839 : 1,454
CDSs length (mean : median) Restricted to multi-exon genes	1,356 : 1,071	1,921 : 1,548
Cumulative size of coding sequences (%)	25,230,147 (8.8%)	26,681,223 (16.1%)
Number of introns	67,414	60,774
Intron length (mean : median)	1,465 : 55	1,446 : 53
Percentage of contigs with >= 1 gene (% in bases)	82.9% (99.2%)	18.5% (97.6%)
BUSCO with Insecta database (N = 1,367)		
Complete	1,319 (96.5%)	1,361 (99.6%)
Duplicated	8 (0.6%)	339 (24.8%)
Fragmented	12 (0.9%)	3 (0.2%)
Missing	36 (2.6%)	3 (0.2%)

**Figure 1.**
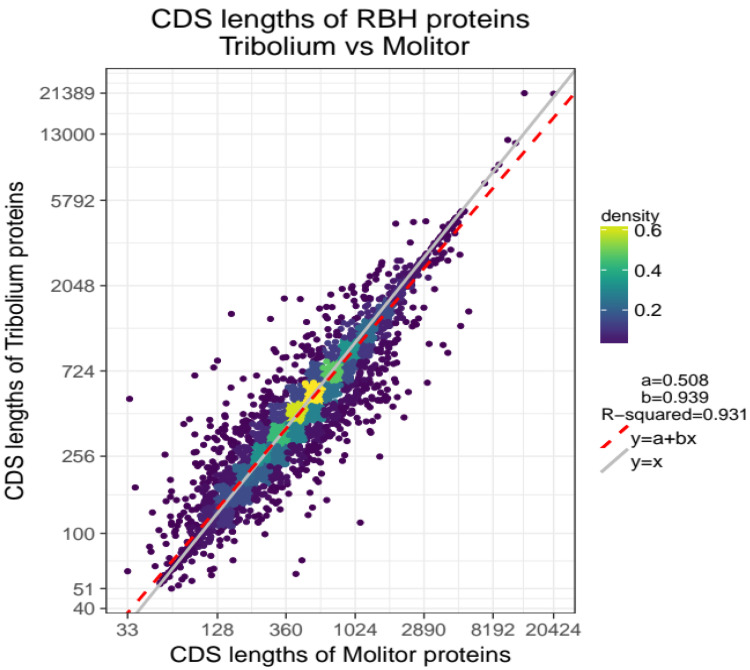
CDS length association for 10,495 orthologous genes of *T. molitor* and *T. castaneum*. Comparison plot with CDS lengths of *T. molitor* on x-axis and CDS lengths of *T. castaneum* on y-axis. Lengths (points) are log-scaled and coloured based on their density (highest density= yellow, lowest density=dark violet). The linear regression model best fitting the data is represented by the red-dashed line *y* = *a* + *bx* with parameters a=0.508 and b=0.939. Higher densities are observed in the central part of the cloud and along the red-dashed fitted regression line.

Not surprisingly, the two species share similar characteristics in terms of CDS lengths and number of exons (Figure S3, *Extended data*) as illustrated in a linear regression model with *R* ^2^=0.931 ( Figure 1).

After stringent gene prediction filtering (see Methods section), the gene structure patterns remained enriched in single-exon genes 22% (compared to 7% of *T. Castaneum*) ( Table 2). Interestingly, 85% of them have a pfam or BlastP hit (evalue=10e-5), suggesting that they are *bona fide* gene predictions. Preliminary results show the existence of paralogous genes among them.

### Genes and repeats evolution in the *Tenebrio molitor* genome

**Figure 2.**
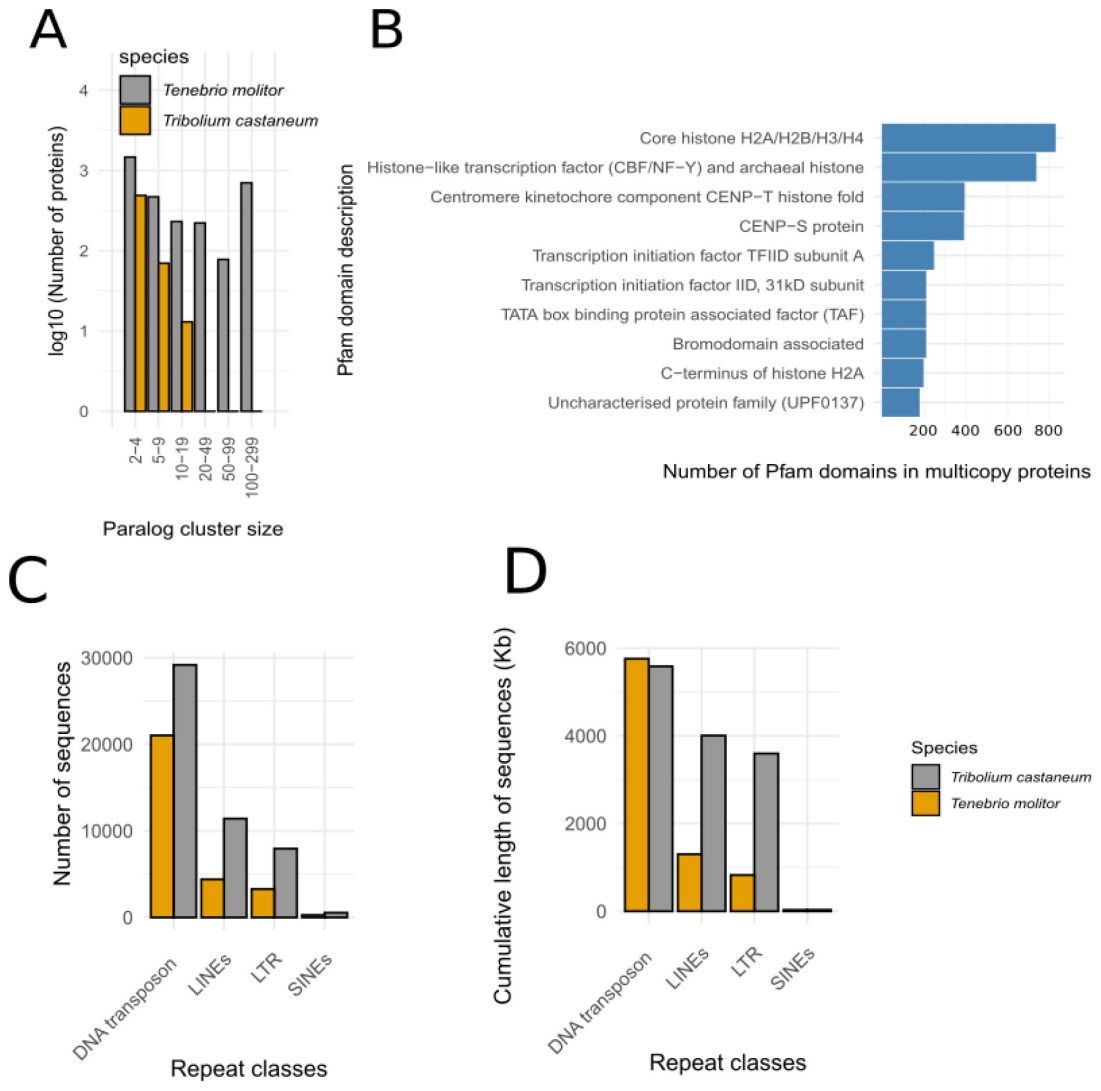
Paralog and repeated sequences analysis between *T. molitor* and *T. castaneum*. **A**. Number of paralogs (log scale) found in the *T. molitor* and *T. castaneum* paralog clusters. **B**. Functional annotation of the *T. molitor* paralogs found in the top 10 largest clusters. **C**. Number of major transposons in the *T. molitor* and *T. castaneum* genome. **D**. Cumulative length of major transposons the *T. molitor* and *T. castaneum* genome.

Paralogs search in the *T. molitor* and *T. castaneum* proteomes revealed a higher proportion of paralogs in *T. molitor* ( Figure 2A). Their functional analyses through Pfam domain annotation showed the overabundance of histone-coding genes organized in blocks located in 25 different scaffolds ( Figure 2B). As an example, one of these scaffolds (scaffold_25 ~545Kb) contains 108 genes coding for histones over its 162 predicted genes. Moreover, the global analysis of histone-coding genes in *T. molitor* showed that 806 genes coding for histones were mono-exonic. Taken together, these results showed that the genome of *T. molitor* has experienced several duplications of mono-exonic histone-coding genes that may also explain the genome size difference between *T. molitor* and *T. castaneum*. However, as the technologies and methods used to produce and annotate the genome of *T. castaneum* were not the same, we cannot ensure that this histone evolution is specific to *T. molitor* or shared among Tenebrionidae.

While DNA transposons are about 50% more abundant in *T. molitor*, they represent about a similar cumulative length ( Figure 2C and B, Supplementary Table 4). On the opposite, the LINEs, SINEs and LTR transposons are about two to three times more abundant in *T. molitor* and their cumulative size is correlated to their abundance. However, in both genomes, the total length of repeated elements represents only 5 to 6% of the genome assembly (Supplementary Table 4).

### Macrosynteny between the *Tenebrio molitor* and *Tribolium castaneum* genomes

**Figure 3.**
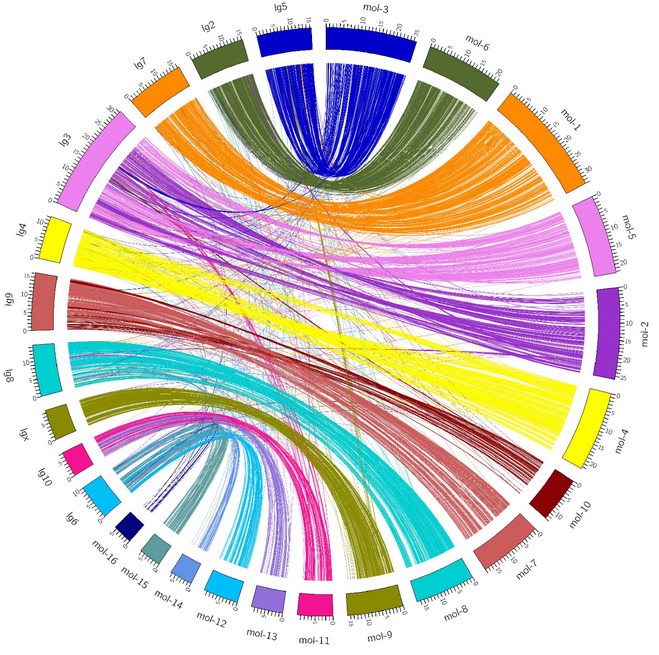
Synteny between *T. molitor* and *T. castaneum*. In the right semi-circle, the longest 16 *T. molitor* scaffolds are represented by orthogonal curved blocks placed next to each other. They are followed by the 10 *T. castaneum* chromosomes (left semi-circle). The unit length of the tick spacing of the blocks is 1Mb so that each block is proportional to the real size of a scaffold/chromosome. The 9,760 protein reciprocal best matches are drawn with colorfoul arches linking the orthologous regions between the two species.

The macrosynteny between the *T. molitor* scaffolds and *T. castaneum* chromosomes ( Figure 3) showed a strong conservation of the genome. The current *T. molitor* assembly lacks the integration of genetic data and linkage groups to reconstruct the entire chromosomes, and the assembly remains fragmented which makes the detection of possible chromosome rearrangements impossible. Future genetic works on *T. molitor* leading to the construction of a high-density map will greatly help to anchor the current assembly on linkage groups to obtain the complete two-dimensional chromosome organisation.

## Conclusions

Our sequencing and assembly strategy to build the heterozygous genome of *T. molitor* by combining long read and Hi-C showed its efficiency and provided a high-quality genome assembly and the first genome annotation with a high completeness. Thanks to this new genomic resource, future work focusing on population, quantitative and functional genomics of genes of interest will be facilitated and will greatly improve our knowledge on the molecular basis of the *T. molitor* biology. Duplication of histones has been well described in many genomes, but here the number of duplications might be one of the highest described The presence of a relatively large number of monoexonic histone-coding genes supported by transcripts and conserved protein domains constitutes a field of investigation to understand the biological role and the evolution of these genes. The comparison of *T. molitor* to other available Coleoptera genomes will also aid better understanding of Coleoptera evolution and diversification. Additionally, thanks to the availability of the *T. molitor* genome and genes, new breeding programs can take advantage of this resource to improve and optimize mealworm production at the industrial scale through the combination of phenotypes and whole-genome genotypes to perform genome-wide association studies, quantitative trait locus analyses and genome estimation breeding values analysis.

## Data availability

### Underlying data

European Nucleotide Archive: Chromosome-scale assembly of the yellow mealworm genome. Accession number PRJEB44684.

European Nucleotide Archive: Chromosome-scale assembly of the yellow mealworm genome. Accession number PRJEB44703.

European Nucleotide Archive: Chromosome-scale assembly of the yellow mealworm genome. Accession number PRJEB44755.

NCBI BioProject: Mater immunity, reference transcriptome of *Tenebrio molitor*. Accession number PRJNA646689.

Other underlying data for the tenebrio genome are available on GitHub and Zenodo.

Zenodo: madoui/Tenebrio_Genome: updated supp data. https://doi.org/10.5281/zenodo.5499691 ^46^.

This project contains the following underlying data:

- Supplementary_Data.pdf / Supplementary Table 6: Samples’ accession numbers.
- Data / monoexonic (BED file with coordinates of monoexonic genes)
- Data / repeat (BED file with coordinates of the repeats)

### Extended data

All extended data are available on GitHub and Zenodo.

Zenodo : madoui/Tenebrio_Genome: updated supp data. https://doi.org/10.5281/zenodo.5499691 ^46^.

This project contains the following extended data within the file ’Supplementary_Data.pdf’:

- Supplementary Table 1: Genomic data
- Supplementary Table 2: Transcriptomic data
- Supplementary Table 3: Metrics for long reads, contigs and scaffolds through different steps
- Supplementary Table 4: Repeats
- Supplementary Note 2: Gmove
- Supplementary Figure 1: GenomeScope Profile for *T. molitor*
- Supplementary Figure 2: K-mer plot before and after Haplomerger
- Supplementary Figure 3: Comparison of CDS lengths and number of exons of orthologous genes between *T. molitor* and *T. castaneum*
- Supplementary Figure 4: Aligning *T. molitor* to *T. castaneum*
- Supplementary Figure 5: Position of the 142 bp satellite (TMSATE1) on scaffolds 16, 58, 99, 23 and their coverage by Illumina Reads
- Supplementary Figure 6: Presence of mitochondrial genome on scaffold 94
- Supplementary Table 5: Alignment between the mitochondrial genome and the scaffold 94
- Supplementary Figure 7: Presence of mitochondrial genome on scaff 65
- Supplementary Figure 8: Alignment of scaffolds 94, 65
- Supplementary Figure 9: Coverage of scaffolds 65, 94 by Illumina mitochondrial reads
- Supplementary Figure 10: Assembly workflow
- Supplementary Figure 11: Annotation workflow

This project also contains the following extended data:

- assembly_workflow.pdf (details of the genome assembly method)
- annotation_workflow.html (details of the genome annotation method)

Data on GitHub and Zenodo are available under the terms of the Creative Commons Zero "No rights reserved" data waiver (CC0 1.0 Public domain dedication).
