# Supplementary material for: Chromosome-scale assembly of the yellow mealworm genome
Source: Open Res Eur. 2022 Sep 5;1:94. Originally published 2021 Aug 17. [Version 3] doi: 10.12688/openreseurope.13987.3 (PMC10445852; doi:10.12688/openreseurope.13987.3)
Supplement: Peer review history

# Peer review: Reviewer response for version 3

**DOI:** 10.21956/openreseurope.16289.r30028

**Published:** 2022-09-05

**Authors:** Gregor Bucher

**Affiliations:** 1 Johann Friedrich Blumenbach Institute, GZMB, University of Göttingen, Göttingen, Germany

The authors have addressed all my concerns and I congratulate them for this paper!

 I have to confess that "chromosome scale" still seems a bit of an overstatement to me but that may be a matter of personal opinion.

Is the study design appropriate and does the work have academic merit?

Yes

Is the work clearly and accurately presented and does it cite the current literature?

Partly

If applicable, is the statistical analysis and its interpretation appropriate?

Not applicable

Are all the source data underlying the results available to ensure full reproducibility?

Yes

Are the conclusions drawn adequately supported by the results?

Partly

Are sufficient details of methods and analysis provided to allow replication by others?

Yes

Reviewer Expertise:

Gene function studies in beetles; evolution and development of the insect head and brain; RNAi in pest control

I confirm that I have read this submission and believe that I have an appropriate level of expertise to confirm that it is of an acceptable scientific standard.

# Peer review: Reviewer response for version 2

**DOI:** 10.21956/openreseurope.15728.r28846

**Published:** 2022-04-13

**Authors:** Gregor Bucher

**Affiliations:** 1 Johann Friedrich Blumenbach Institute, GZMB, University of Göttingen, Göttingen, Germany

Eleftheriou et al. present their work on enhancing the genomic sequence and annotation of the yellow mealworm *Tenebrio molitor*, an insect species potentially providing an alternative source for feed and food. Based on extensive sequencing of DNA and RNA and by using long- and short read technology and Hi-C data they provide a very much-improved version of the genomic sequence and gene set of this species (e.g. dramatically increasing N50). Finally, they assess aspects of genome assembly and gene set by comparing with another well-sequenced beetle, *Tribolium castaneum*.

**Questions with respect to the analyses:**

 1. You compare your gene set and genome to *T. castaneum*. What assembly and gene set did you use for that purpose? Richards et al ^1^., Herndon et al ^2^., NCBI, …? For example, I could e.g. not match your N50 values (table 1) or gene numbers (table 2) to those published in the latest genome paper (Herndon et al ^2^). Please provide references and explain, where you got your numbers from.

 2. The higher number of annotated genes in *T. molitor* compared to *T. castaneum* is potentially interesting but-in my opinion-not yet sufficiently supported. It could be interesting biology or annotation artifact (some numbers in table 2 would be in line with the latter assumption: shorter transcripts with less exons but more single exon genesin *T. molitor*. It could for instance have happened during the processing step to split erroneously assembled transcripts). Some suggestions on alternative approaches for annotation/splitting transcripts in order to test, whether the number really is that different:

- If I understood correctly, you first assembled the transcriptome from RNA-seq data only and used algorithms to split potentially fused transcripts. While I find the given criteria convincing, the number of splits still depends on the parameters that you choose. Why not use your new and excellent genome sequence information for that purpose? If two parts of a predicted transcript map in close proximity to each other and in the right orientation on the same scaffold, it should probably not be split.
- A more fundamental question: Why not first map the RNA-seq reads onto your genome assembly and then apply annotation tools?
- A very good criterion to fuse or to split predictions are “split reads” (i.e. RNA-seq reads that span intron boundaries and therefore map to different exon boundaries on the genome thereby precisely mapping the intron).

3. Strongly increased paralog cluster size. This finding seems unexpected and interesting but again I would like to see some further analyses to confirm this. The increase is specifically striking with respect to the large cluster sizes (> 20 copies) where *T. castaneum* does not seem to have any cluster but *T. molitor *many. Your analysis in Fig. 2B seems based on all clusters - it would be nice to manually follow-up some of the intriguing large clusters in order to confirm that they are real and to understand the underlying pattern. Focused on the large clusters questions could be: What protein families are heavily expanded (apart from histones)? Where in the genome are the paralogs of selected expanded gene families located (clustered or evenly distributed, do they map to the chromosome parts that seem ambiguous in their syntenty to *Tribolium*?). If they are enriched in ambiguous genome regions – would that indicate assembly problems or localized genome changes? The results should either confirm this intriguing finding and allow for hypotheses on the underlying patterns or they might reveal that this reflects annotation bias.

 4. Genome size difference: The overall genome size is different and I would have appreciated some words on what the explanation is. More ORFs? Paralog expansion? Repetitive sequences (you mention that in both genomes that is around 5-6%). You mention that the histone expansion may explain this – how much (in %) does this gene family contribute to the expansion – base on that number: is it one of many drivers or one of the key drivers? What are the other drivers?

 5. BUSCO: you provide two different BUSCO analyses in table 1 vs. table 2. Either you remove the one that you consider less precise or you explain what the difference in the analyses is and what it means.

 6. Synteny: for most scaffolds, it seems quite clear to what chromosome of *Tribolium *they correspond – either individually or fused with other scaffolds (based on Fig. 3). LG6 seems to be split into several – any explanation for that? Do you expect to have assembled the Y chromosome (given that you used a male) – how does that compare with *Tribolium*? Given that you know the number of expected chromosomes in your species, some thoughts on which scaffolds probably belong to one chromosome would be nice.

**Typos and minor comments:**

**Title:**

 While the authors provide an excellent new assembly I agree with the first reviewer that the term “chromosome-scale” may seem a bit strong given that based on latest technology, some of the currently emerging insect genomes reflect the expected number of chromosomes almost completely.

**Plain language summary:**

 “red flour beetle”

**Methods:**

 The metamorphic stage should be called pupa (the term nymph seems to be reserved for the pre-adult stages in hemimetabolous insects) – please replace (3 times at least).

 I suggest using “embryos” or “embryonic stages” instead of “eggs”

 “… with chloroform centrifugation….” Did you mean extraction?

 RNA extraction: Please specify temperature and the time window of egg collection as this determines the embryonic stages that are covered.

 “… occur in the same orientation as antisense RNA.”  - maybe more clear “…occur in antisense orientation only.”

 “cDNA was then 3’-adenylated…”

 Transcriptome assembly: “…developmental stages (embryos, larvae, pupae adults)”

 “…and only contigs larger than 150…” – did you mean “reads” here? Same issue two lines below.

 “… this tool aims to split contigs with different functional sites into different contigs.” – is this what you meant?

**Results:**

 “ 7% of *T. Castaneum*…”

 “… we cannot ensure that this expansion of histone genes is specific to …”

 There are more DNA transposons but the overall length is similar – does that mean that they are shorter?

Is the study design appropriate and does the work have academic merit?

Yes

Is the work clearly and accurately presented and does it cite the current literature?

Partly

If applicable, is the statistical analysis and its interpretation appropriate?

Not applicable

Are all the source data underlying the results available to ensure full reproducibility?

Yes

Are the conclusions drawn adequately supported by the results?

Partly

Are sufficient details of methods and analysis provided to allow replication by others?

Yes

Reviewer Expertise:

Gene function studies in beetles; evolution and development of the insect head and brain; RNAi in pest control

I confirm that I have read this submission and believe that I have an appropriate level of expertise to confirm that it is of an acceptable scientific standard, however I have significant reservations, as outlined above.

## References

[1] : The genome of the model beetle and pest Tribolium castaneum. *Nature* .2008;452(7190) : 10.1038/nature06784 949-55 10.1038/nature06784 18362917

[2] : Enhanced genome assembly and a new official gene set for Tribolium castaneum. *BMC Genomics* .2020;21(1) : 10.1186/s12864-019-6394-6 47 10.1186/s12864-019-6394-6 31937263PMC6961396

# Response

**Published:** 2022-08-24

**Authors:** Mohammed-Amin Madoui

**Affiliations:** 204936, France

Eleftheriou et al. present their work on enhancing the genomic sequence and annotation of the yellow mealworm Tenebrio molitor, an insect species potentially providing an alternative source for feed and food. Based on extensive sequencing of DNA and RNA and by using long- and short read technology and Hi-C data they provide a very much-improved version of the genomic sequence and gene set of this species (e.g. dramatically increasing N50). Finally, they assess aspects of genome assembly and gene set by comparing with another well-sequenced beetle, Tribolium castaneum.  

 Questions with respect to the analyses:

**Question 1:** You compare your gene set and genome to *T. castaneum*. What assembly and gene set did you use for that purpose? Richards et al1., Herndon et al2., NCBI, …? For example, I could e.g. not match your N50 values (table 1) or gene numbers (table 2) to those published in the latest genome paper (Herndon et al2). Please provide references and explain, where you got your numbers from.

**Answer 1:** Indeed the reference of the genome version used for comparative genomics was missing. We used the Herndon et al version. We added the reference in the manuscript. The genome assembly and gene annotation metrics were calculated directly from the data downloaded from NCBI https://www.ncbi.nlm.nih.gov/genome/216.

**Question 2.1:** The higher number of annotated genes in *T. molitor* compared to *T. castaneum* is potentially interesting but in my opinion-not yet sufficiently supported. It could be interesting biology or annotation artifact (some numbers in table 2 would be in line with the latter assumption: shorter transcripts with less exons but more single exon genesin *T. molitor*. It could for instance have happened during the processing step to split erroneously assembled transcripts).  Some suggestions on alternative approaches for annotation/splitting transcripts in order to test, whether the number really is that different:

 • If I understood correctly, you first assembled the transcriptome from RNA-seq data only and used algorithms to split potentially fused transcripts. While I find the given criteria convincing, the number of splits still depends on the parameters that you choose. Why not use your new and excellent genome sequence information for that purpose?

  If two parts of a predicted transcript map in close proximity to each other and in the right orientation on the same scaffold, it should probably not be split.

**Answer 2.1:** Although the splitting method is based on several parameters, it splits only 5% of the contigs. And 60% of the contigs split in two were oppositely oriented (orientation is inferred by RNA-reads alignment). The rest (40% of split contigs) with the same orientation were split because of a significant difference in coverage (based on the mpileup of RNA reads) between the two sides of the break point. Considering the proportion of split transcript contigs, we think that the high number of single-exon genes in *T. molitor* is not produced during the splitting step, but reveals interesting biology in the *Tenebrio molitor* genome (now presented in the manuscript). Here is a typical example of a contig split into two parts: See figure 1 here.

 • The track, named “Gmove Final 21435 genes”, shows two different genes. They are two co-oriented tandem duplicated genes.

 • All RNA-seq contigs (purple tracks) show two distinct genes.

 • What strengthens the necessity of splitting are the protein mappings (green tracks). None of these alignments covers the two loci with all the exons. • For *T. castaneum* the same protein aligns twice in a row («esterase FE4 isoform X1 on the left and «esterase E4» on the right.

 • Also, Gmove predicts two genes spanning all exons based on the split contigs.

 Here is another example of a tandem single-exon gene is the leucine-rich repeat-containing protein of the figure 2 here.  

 • At first, the transcripts are assembled with Oases. However, Oases does not take into account the orientation (as other assemblers do, eg Trinity). So, it makes sense to look for RNA contigs erroneously assembled.

 •Subsequently, reads that are not properly paired are eliminated. Then, depending on the depth of coverage, potential breakpoints are defined.

 •We finally validate the breakpoints based on ORFs (Transdecoder) and domains (CDDsearch) information.  

**Question 2.2:** A more fundamental question: Why not first map the RNA-seq reads onto your genome assembly and then apply annotation tools?

**Answer 2.2:** This is, indeed, another way to perform the annotation, although it does not guaranty more robust results. We could have used, for instance, hisat2 combined with StringTie in order to align RNA-seq reads onto the genome and then assemble. However, it was not a priority to test every possible methods of annotation.      

**Question 2.3:** A very good criterion to fuse or to split predictions are “split reads” (i.e. RNA-seq reads that span intron boundaries and therefore map to different exon boundaries on the genome thereby precisely mapping the intron).

**Answer 2.3: **Yes, split reads are indeed good predictors to fuse or split predictions when you work with read alignments but in our case we work with contigs alignment so we use “split contigs” to define intron boundaries, which are also good predictors and  similar to the ”split reads” approach.    

**Question 3.1:** Strongly increased paralog cluster size. This finding seems unexpected and interesting but again I would like to see some further analyses to confirm this. The increase is specifically striking with respect to the large cluster sizes (> 20 copies) where *T. castaneum *does not seem to have any cluster but *T. molitor* many. Your analysis in Fig. 2B seems based on all clusters - it would be nice to manually follow-up some of the intriguing large clusters in order to confirm that they are real and to understand the underlying pattern. Focused on the large clusters, questions could be: What protein families are heavily expanded (apart from histones)?  

**Answer 3.1:** It is a very interested question. To address it, we added the following table in the main manuscript (now table 3) that illustrates the biology behind the gene duplications. See table 1.

**Question 3.2:** Where in the genome are the paralogs of selected expanded gene families located (clustered or evenly distributed, do they map to the chromosome parts that seem ambiguous in their syntenty to Tribolium?).  

**Answer 3.2:** As for histones, most of the duplicated genes are organized in small clusters randomly distributed in large scaffolds of the genome but for the antifreeze proteins that are all located in a single large cluster. This information is now mentioned in the manuscript.  

**Question 3.3: **If they are enriched in ambiguous genome regions – would that indicate assembly problems or localized genome changes? The results should either confirm this intriguing finding and allow for hypotheses on the underlying patterns or they might reveal that this reflects annotation bias.  

**Answer 3.3:** Manual curation showed that the duplicated genes clusters are located in gene rich regions of large scaffolds which support the presence of many duplication events in Tenebrio. Except for the antifreeze proteins that are located in a single cluster on the scaffold 7. This information is now mentioned in the manuscript.  

**Question 4:** Genome size difference: The overall genome size is different and I would have appreciated some words on what the explanation is. More ORFs? Paralog expansion? Repetitive sequences (you mention that in both genomes that is around 5-6%). You mention that the histone expansion may explain this – how much (in %) does this gene family contribute to the expansion – base on that number: is it one of many drivers or one of the key drivers? What are the other drivers?  

**Answer 4:** This is a difficult question to address and we have tried to give some new elements. We identified one repeat element of 77kb present 318 times in the *Tenebrio molitor* genome and absent in Tribolium. The cumulative size of this element leads to 20Mb of sequences. On the other side, the histone gene sequences correspond to 7.4Mb and the cumulative size of the gene famlies listed in the table 3 lead to 16.3Mb. By taking into account these numbers, we see that both gene duplication and repeated element participated to the genome expansion. Also by looking at the circus plot (Figure 3), we see that the large scaffolds anchoring on Tribolium linkage groups are larger than the Tribolium chromosomes but for the LG8. This shows that the expansion is quite homogeneous in the different Tenebrio chromosomes and involved both genes and repeats expansion.  

**Question 5:** BUSCO: you provide two different BUSCO analyses in table 1 vs. table 2. Either you remove the one that you consider less precise or you explain what the difference in the analyses is and what it means.  

**Answer 5: **Indeed, the BUSCO analysis on the genome assembly reflects the completeness of the genome assembly while the BUSCO analysis on the genome annotation reflects the completeness of the gene prediction. This has been mentioned as follow “The published gene prediction based on the *T. castaneum* genome has fewer genes but a higher BUSCO score, which reflects the completeness of the gene prediction while the BUSCO score on the genome assembly reflects the completeness of the genome assembly. »  

**Question 6.1:** Synteny: for most scaffolds, it seems quite clear to what chromosome of Tribolium they correspond – either individually or fused with other scaffolds (based on Fig. 3). LG6 seems to be split into several – any explanation for that?

**Answer 6.1: **We can explain the presence of two large scaffolds anchoring on the LG6 of Tribolium by a lack of data allowing joining the two scaffolds. More long-range information provided by long reads, Hi-C or optical maps and the use of the Tenebrio genetic map will help to mind this gap.

**Question 6.2:** Do you expect to have assembled the Y chromosome (given that you used a male) – how does that compare with Tribolium?

**Answer 6.2: **Indeed, we expect to have assembled the Y chromosome but the investigation of the male specific genes is still undergoing. The Tribolium sequence of the Y chromosome was not provided in our data. A high-density genetic map of Tenebrio will be soon constructed and will help to identify the scaffolds corresponding to the Y chromosomes. Also, future work on male specific gene expression ill also help to identify Y chromosomes genes.

**Question 6.3:** Given that you know the number of expected chromosomes in your species, some thoughts on which scaffolds probably belong to one chromosome would be nice. **Answer 6.3: **We are currently building a genetic map for *T. molitor* that will allow us to properly anchor our genome assembly on the chromosomes. This will also help to characterize the Y chromosome. We plan to release the pseudo-molecules after the complete analysis of the genetic data.  

 Typos and minor comments:

**Comment 1:** Title: While the authors provide an excellent new assembly I agree with the first reviewer that the term “chromosome-scale” may seem a bit strong given that based on latest technology, some of the currently emerging insect genomes reflect the expected number of chromosomes almost completely.

**Answer:** We understand your opinion concerning the title. We obtained one or two very large scaffolds for each chromosome (according to the synteny with *T. castaneum*) except for one chromosome corresponding to the linkage group 6 in *T. castaneum* where we obtained four large scaffolds. Thus, we used the term “chromosome-scale” because the scaffolds have the chromosome length scale i.e several megabases. We distinguish the term “chromosome-scale”, commonly used when you obtain very large scaffolds reaching the chromosome size, to the “telomere-to-telomere” term which is used when you obtain the complete chromosome sequences without gaps.  

**Comment 2:** Plain language summary: “red flour beetle”  

**Answer:** This has been corrected  

**Comment 3:** Methods: The metamorphic stage should be called pupa (the term nymph seems to be reserved for the pre-adult stages in hemimetabolous insects) – please replace (3 times at least).  

**Answer:** all the occurrences has been corrected  

**Comment 4:** I suggest using “embryos” or “embryonic stages” instead of “eggs”  

**Answer:** This has been modified  

**Comment 5:** “… with chloroform centrifugation….” Did you mean extraction?

**Answer:** Indeed, we meant extraction, this has been changed  

**Comment 6:** RNA extraction: Please specify temperature and the time window of egg collection as this determines the embryonic stages that are covered.

**Answer: **The eggs were collected at RT and collected within a week after laying. This is now mentioned in the manuscript.  

**Comment 7** “… occur in the same orientation as antisense RNA.”  - maybe more clear “…occur in antisense orientation only.”

**Answer:** This has been modified according to your advice  

**Comment 8:** “cDNA was then 3’-adenylated…”

**Answer:** This has been corrected  

**Comment 9:** Transcriptome assembly: “…developmental stages (embryos, larvae, pupae adults)”

**Answer:** This has been corrected  

**Comment 10:** “…and only contigs larger than 150…” – did you mean “reads” here? Same issue two lines below.

**Answer: **We meant “contigs”  

**Comment 11:** “… this tool aims to split contigs with different functional sites into different contigs.” – is this what you meant?

**Answer:** This has been corrected  

**Comment 12:** Results: “ 7% of T. Castaneum…”

**Answer:** This has been corrected  

**Comment 13:** “… we cannot ensure that this expansion of histone genes is specific to …” **Answer:** The sentence has been modified according to your advice  

**Comment 14:** There are more DNA transposons but the overall length is similar – does that mean that they are shorter?

**Answer:** It seems that DNA transposons are possibly shorter in Tribolium compared to Tenebrio. This observation goes in the sense of a genome expansion in Tenebrio compared to Tribolium in which DNA transposons may have participated.  

*We gratefully thank the reviewer for its very helpful comments and suggestions.*

# Peer review: Reviewer response for version 2

**DOI:** 10.21956/openreseurope.15728.r28658

**Published:** 2022-04-04

**Authors:** Barbara Feldmeyer

**Affiliations:** 1 Senckenberg Biodiversity and Climate Research Centre, Frankfurt am Main, Germany

I have read the revised version of the manuscript, and response to reviewer. The authors did a good a job in revising the manuscript, and I have no further comments.

Is the study design appropriate and does the work have academic merit?

Yes

Is the work clearly and accurately presented and does it cite the current literature?

Yes

If applicable, is the statistical analysis and its interpretation appropriate?

Yes

Are all the source data underlying the results available to ensure full reproducibility?

Yes

Are the conclusions drawn adequately supported by the results?

Partly

Are sufficient details of methods and analysis provided to allow replication by others?

Yes

Reviewer Expertise:

Evolutionary biology, genomics, transcriptomics

I confirm that I have read this submission and believe that I have an appropriate level of expertise to confirm that it is of an acceptable scientific standard.

# Peer review: Reviewer response for version 1

**DOI:** 10.21956/openreseurope.15073.r27973

**Published:** 2021-11-24

**Authors:** Barbara Feldmeyer

**Affiliations:** 1 Senckenberg Biodiversity and Climate Research Centre, Frankfurt am Main, Germany

The authors construct a new “chromosome” assembly of the T. molitor genome using current state of the art technology. While the manuscript is well written and clear in most parts there are three major aspects which should be addressed before indexing of the manuscript.

**General major comments**

1. I find the title = chromosome scale somewhat misleading given the information later in the text. The authors obtain 112 scaffolds using the HiC data but then discuss 16 scaffolds representing 90% of the genome. Do you know/expect T.molitor to have 16 chromosomes? I could not find any information on that matter. Is the conclusion of having a chromosome scale assembly justified and why? Please add this information in the discussion section for example.
2. The comparative genomic section consists of a Blastp of both protein sets and a synteny plot. I understand that genome re-arrangement analyses are not feasible, but what about the gene content. Which genes or gene families are found or maybe enriched in the +5000 genes in T.molitor? How do repeat contents differ between species?
3. The mitochondrial sequence seems to be attached to 2 (3?) scaffolds, where it should be a single circular sequence only. This indicates some extent of misassembly and should at least be manually curated.

**Introduction**

- The global population: specify that you are talking about the HUMAN not beetle population.
- “However, prior genomic resources on *T. molitor *are needed to accelerate such genetic programs.”: Not sure that “prior” is the right wording here. Maybe “suitable”, or “ a good quality genomes”…
- Change “Here, we propose a *T. molitor *genome assembly based” to “Here, we present”
- “kept at room temperature and humidity”: % humidity information is missing.
- Change “put on a starvation for three days”  to “ were starved for three days”

**Methods**

- “For mRNA extraction, eggs, larva, nymphs, adult males and females were isolated without specific diet”: More information needed. Did you make one extraction from all samples, did you isolate each sample individually, did you sequence one pool or each sample individually, which kit did you use for extraction, did you Illumina sequence and which depth? => after reading further I realize that this information is given later. Maybe rephrase the sentence to “Eggs, larva, nymphs, adult males and females were collected for later mRNA extraction.”
- “HMW gDNA was size-selected using Short Read Eliminator” change to “…using the Short…”
- “following protocol, using the Oxford Nanopore” remove “using”.
- Dovetail “The one-third of the cryoground” change to “Another third of the ….”
- “obtained 138 scaffolds. Only scaffolds larger than 35kb were kept resulting in a final assembly of 112 scaffolds” why >35kb?
- Transcriptome “The contigs 5’ and 3’ends were cleaned.” Using which tool and parameters?
- Why did you use Velvet and Oases especially since they are prone to create chimeras? Why not use Trinity for example?
- Change “the research of ORFs” to “the identification of ORFs”.
- Change “aims to split contigs sequences” either to “aims to split contigs with different…” or “aims to split contig sequences”
- Do you make the contig splitting tool available somewhere? Is the code accessible on github or in the supplement? If so add reference.
- “BLAT (version 36 with default parameter) matches with score” change to “with a score”

**Results and Discussion**

- Table 1 compares the newest version of Tmolitor with the older and Tcastaneum but on contig scale. Why contig and not scaffold, or chromosome scale?
- “of 99.5% (using version 5.0.0 with Insecta database odb10) while the predicted genome size was 310 Mb (Figure S1, *Extended data*)” remove “while the predicted genome size was 310 Mb (Figure S1, *Extended data*)” from this sentence. You give this information at the beginning of the paragraph already.
- "Furthermore, mitochondrial genome“ change to “ “the mitochondrial genome”.
- “More precisely, the mitochondrial genome of *T. molitor *(15,785 bp) was aligned to the assembly using Minimap245.”: This whole mt paragraph is confusing. Do you mean you aligned an existing mt genome, from the previous assembly? From which other study? => add reference and/or accession number. And you aligned it to identify the mt genome in your assembly. Why is the mt genome split across scaffolds? It should be on single contig only? => It sounds like misassembly and should be corrected. I.e. the mt parts should be assembled into a separate mt genome and should be removed from the scaffolds.
- “a total of 21,435 genes which is higher than the number observed in *T. castaneum*”: could you discuss why you think you have more genes but lower Busco (Table2)?

**Conclusions**

- Change “future works” to “future work”.

Is the study design appropriate and does the work have academic merit?

Yes

Is the work clearly and accurately presented and does it cite the current literature?

Yes

If applicable, is the statistical analysis and its interpretation appropriate?

Yes

Are all the source data underlying the results available to ensure full reproducibility?

Yes

Are the conclusions drawn adequately supported by the results?

Partly

Are sufficient details of methods and analysis provided to allow replication by others?

Yes

Reviewer Expertise:

Evolutionary biology, genomics, transcriptomics

I confirm that I have read this submission and believe that I have an appropriate level of expertise to confirm that it is of an acceptable scientific standard, however I have significant reservations, as outlined above.
